# Internationalization of family firms on innovation: Moderating impact of socioemotional wealth and institutional environment

**DOI:** 10.1371/journal.pone.0322343

**Published:** 2025-05-12

**Authors:** Lixin Zhou, Hanwei Zhou, Xiao Wei

**Affiliations:** Institute for Chengdu-Chongqing Economic Zone Development, Chongqing Technology and Business University, Chongqing, China; Maynooth University, IRELAND

## Abstract

Currently, there is a lack of research specifically investigating the influence of internationalization on the innovation of family firms. This paper aims to investigate the influence of both internationalization depth and breadth on the innovation of family firms. Additionally, it aims to analyze how socioemotional wealth (SEW) and regional institutional environment in the home country influence this relationship. To test the hypotheses, a questionnaire survey was carried out with a sample of 253 family firms situated in eight provinces or municipalities in China. The results are as follows: First, internationalization depth negatively influenced family firm innovation, whereas internationalization breadth positively influenced family firm innovation. Second, the negative influence of internationalization depth on innovation was pronounced at a high level of family control, whereas the positive influence of internationalization breadth on innovation was pronounced at a low level of transgenerational succession intention in family firms. Third, a well-developed institutional environment strengthened the relationship between internationalization and innovation in family firms. This research enriches the literature on internationalization and innovation in family firms, and offers useful insights for practitioners.

## Introduction

Since China’s reform and opening up over 40 years ago, private enterprises have played an important role in driving economic development, creating jobs, promoting technological innovation, and raising tax income. They account for more than half of tax income, more than 60% of GDP, over 70% of total technology innovation, and more than 80% of urban employment. More over 80% of China’s private enterprises are family businesses [1].

China advocates for an innovation-driven development strategy for the future. With the implementation of China’s “going abroad” strategy, Chinese family firms have gradually advanced the pace of internationalization in recent years. According to PricewaterhouseCoopers’ 2014 Family Business Survey Report, international sales were 15% of overall revenues for Chinese mainland family firms in 2014. Furthermore, the 2016 Family Business Survey Report found that nearly 80% of Chinese mainland family firms exported goods or services to international markets in 2016.

Internationalization and innovation are considered crucial strategies for obtaining and maintaining the firm competitive advantage which drives family firm’s long-term survival and sustainable growth [2]. During the internationalization process, family firms are exposed to new market experiences and other knowledge, which provides opportunities for innovation and development [3]. Therefore, family firms must consider innovation as a crucial aspect while expanding their operations internationally.

Currently, the research on family firm internationalization focuses on the influencing factors, process, and performance of internationalization [4–6]. However, few studies analyze the impact of internationalization on innovation in family firms. Furthermore, the limited research available only investigates the impact of internationalization depth (i.e., export intensity) on the innovation of a family firm [7–9], ignoring the impacts and differences of both internationalization depth and breadth on family firm innovation. This paper argues that further research is needed in this field. Internationalization depth is often conceptualized as the extent of its foreign operations and investments, whereas internationalization breadth is defined as the firm’s scope of international operations [10,11].

Socioemotional wealth (SEW) refers to the affective endowments or non-financial benefits that family principals obtain from their business [12]. It plays a crucial role in defining the unique characteristics of a family firm and influencing its strategic decisions [13,14]. As such, SEW has the potential to affect family firm internationalization [4,15]. However, there is a lack of research exploring the influence of SEW on the connection between internationalization and innovation in a family firm setting.

The macro-institutional environment plays a crucial role in influencing the internationalization of family firms [16]. During China’s economic transition, significant differences in institutional environment among different regions were observed, thus impacts the connection between internationalization and innovation of a family firm.

This paper aims to explore the impacts of both internationalization depth and breadth on family firm innovation, as well as to investigate the influence of SEW and the home country’s regional institutional environments on the relationship between internationalization and innovation in family firms.

The theoretical contributions of this paper are as follows. First, our study advances the resource-based view by revealing the dichotomous effects of family firms’ internationalization strategies: depth entrenches resource rigidity and path dependence, constraining innovation, whereas breadth enables cross-market learning and resource recombination, fostering innovation. These insights expand our comprehension of the connection between internationalization and innovation in family firms from the resource-based view, in line with Zahra’s [17] request for further research on how internationalization might enhance the innovative capacities of family firms. Second, we analyze the moderating role of SEW on the relationship between internationalization and innovation in family firms, an area that has not been explored in the previous literature [7–9]. We find that transgenerational succession intention weakens the positive influence of internationalization breadth on family firm innovation. Our findings also highlight the possible emergence of a “dark side” of certain SEW dimensions in the context of family firm internationalization [18]. Finally, our studies provide additional empirical evidence on how institutions influence the relationship between internationalization and innovation in family firms.

## Literature review and hypotheses development

### Literature review

#### The relationship between internationalization and innovation of family firms.

The internationalization of family firms has received considerable research attention. Previous research on family firm internationalization has examined affecting factors, processes, and performance [4–6]. Most research assumes that the unique features of family firms influence their internationalization [5], as well as the unique context or institutional features in which their decisions are taken [16]. Some scholars have investigated the impact of innovation on the internationalization in family firms [19–22]. However, few studies have sought to explore the impact of internationalization on innovation in the context of family firms. Several scholars have been using a resource-based theory and relational network theory to argue that internationalization (i.e., export status and intensity) positively promotes the innovation of family firms [7–9]. Based on the relational network theory, Bian et al. [8] discovered that internationalized operation had a positive impact on innovation investment of family firms. Yu et al. [9] proposed that the learning and scale of effects from exporting significantly promoted R&D investment of family firms. Sanchez-Marin et al. [23] suggested that family management promotes the learning-by-exporting effect on product innovation via an inverted U-shaped pattern. The existing study ignores the impacts and variations of internationalization depth (i.e., scale) and breadth (i.e., scope) on family firm innovation.

### Restricted and extended SEW

Gómez-Mejía et al. [12] proposed SEW theory to explain family firms’ distinct behaviors, prioritizing SEW preservation over economic goals. SEW represents non-economic and emotional value associated with a family firm that serves to meet the family’s affective needs [12,14]. SEW is a multi-dimensional construct. Berrone et al. [14] proposed that it comprises family control and influence, emotional attachement of family members, identification with the firm, binding social ties, and the renewal of family bonds through dynastic succession. Miller & Le Breton-Miller [24] further divide SEW into two categories: restricted and extended.

Restricted SEW focuses on short-term, and family-centred goals, such as putting family members in key leadership roles regardless of qualifications to strengthen family control, ensuring long-term job security for family members, using firm resources to resolve family disputes, engaging in unrequited altruism or nepotism, and reinforcing the family’s identification with and attachment to the firm [24,25]. Maintaining family control over the firm is the core dimension of Restricted SEW, with the other dimensions depending on it. The primary reasons are as follows. Firstly, family leaders who emphasize unrequited altruism are more likely to provide indiscriminate job security or utilize the firm resources to offer special favors to family members. This diversion of resources is only possible when the family maintains control over the firm. Secondly, the family’s emotional attachment to the firm intensifies their desire for control. The founding and managing of family firm strengthens the family’s identification with and attachment to the firm, thereby increasing their need for family control [12]. This attachment and identification also foster psychological ownership among family members, leading them to resist non-family control and influence. Therefore, maintaining family control of the firm is central to restricted SEW, fostering the strategic conservatism of family firms.

Extended SEW emphasizes a long-term focus, including enhancing a family’s image and reputation, forming [2] sustaining relationships with internal and external stakeholders, and ensuring transgenerational succession [24,26]. Miller et al. [24] argue that fulfillment of extended SEW depends on the longevity of family firms. Transgenerational succession clearly reflects the family’s long-term focus, prompting family firms to prioritize their longevity [27]. Achieving this often necessitates preserving the family’s image and reputation, balancing the family’s interests with those of stakeholders, and forming sustaining relationships with stakeholders. As a result, transgenerational succession is central to extended SEW, which predisposes family firms to make strategic decisions requiring a long investment horizon [28].

We propose that the influence of SEW on the relationship between internationalization and innovation in family firms may be analyzed through two distinct aspects: the intention for family control, and transgenerational succession.

### Institutional environments in emerging economies

Institutions are commonly recognized as rules, both formal (e.g., regulations, laws) and informal (e.g., codes of conduct, norms), that dictate how firms operate [29]. Institutions occupy multidimensional levels (e.g., national, and regional). At the national level, emerging economics like China maintains authority through a hierarchical appointment structure that assures regional officials follow national policies. At the regional level, economic decentralization gives local governments the autonomy and resources they need to run their economies, encourages them to introduce market-based principles and competition, and competes with other regions. This results in considerable regional disparities in institutional environments, especially in their degrees of marketization, with coastal provinces having a well-developed institutional environment aligned with a market economy, while inland provinces lag behind on these reforms in China [30]. In this paper, we investigate how the regional institutional environment impacts the relationship between internationalization and innovation in family firms.

**Fig 1:**
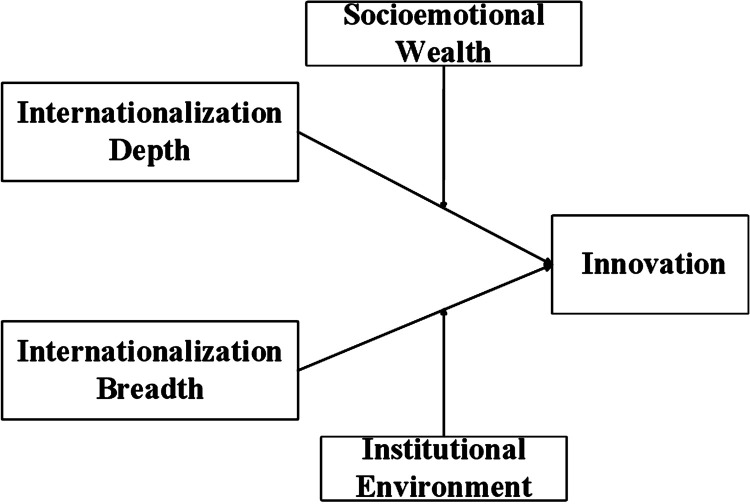
Research framework.

We present our hypotheses in Fig 1.

## Hypotheses development

### Internationalization and family firm innovation

#### Internationalization depth and family firm innovation.

The resource-based theory emphasizes how firms gain a competitive advantage through learning and knowledge accumulation. The firm’s ability to internationalize and interact with overseas partners promotes learning by producing and developing new knowledge, hence prompting innovation [7].

We anticipate that internationalization depth negatively impacts on the innovation of family firms. High depth refers to the situation where family firms operate in a limited number of host countries. Family firms with a high depth gain a comprehensive understanding of tacit market-specific knowledge (e.g., consumer behavior, social networks, regulatory and social institutions), resources and capabilities (e.g., managerial know-how, routines or best practices) embedded in these international markets. However, this reliance on local knowledge creates path dependence, lock-in effect and organizational inertia, making it difficult for family firms to break from existing knowledge frameworks. It also restricts their ability to quickly adapt and transfer knowledge to other international markets, thus limiting their exploration of new knowledge. Additionally, family firms with a high depth may become excessively focused on short-term market feedback and performance targets, neglecting long-term strategic adjustments. This strategic myopia causes family firms to rely excessively on existing knowledge, overlooking opportunities offered by emerging external technologies. As a result of path-dependence, lock-in effects, organizational inertia, and learning myopia [31], family firms with a high depth may face more difficulties in acquiring new knowledge that differs from their existing technological foundation [32]. However, such new knowledge may be highly valuable, scarce, inimitable, and irreplaceable, helping family firms maintain a competitive advantage. In particular, Chinese family firms face such difficulties as they are predominantly involved in labor-intensive manufacturing and traditional services, thus hindering their potential to innovate. Based on the above arguments, we hypothesize:

**Hypothesis 1a**: Internationalization depth has a negative association with the innovation of family firms.

#### Internationalization breadth and family firm innovation.

Internationalization enables firms to interact with other members of the relational network by means of learning, communication, and cooperation. This interaction leads to the acquisition of new information and a heightened motivation to generate new knowledge [33].

We argue that internationalization breadth positively impacts the innovation of family firms. Family firms may face unique challenges in innovative activities due to their restricted resources [34] and limited talent pool [35]. Family firms with a high breadth are more likely to establish extensive partnerships and alliances with foreign companies, allowing them to accumulate valuable, scarce and complementary knowledge from overseas markets through their foreign subsidiaries [36]. They can also profit from knowledge spillovers in overseas marketplaces [11], creating further opportunities for technological collaborations with overseas organizations. Furthermore, high breadth allows family firms to acquire more vast market-specific knowledge, which is highly valuable, scarce, inimitable, and irreplaceable, thus improving their ability to manage market volatility and uncertainty, and consequently reducing the negative influence of market dynamics on innovative activities [32]. Additionally, high breadth implies that firms must adjust their routines and learning paths to swiftly adapt to the new and diverse surroundings [37], resulting in opportunities for innovation [38]. Empirical research suggests the positive impact of internationalization on firm innovation [39]. Based on the above arguments, we hypothesize:

**Hypothesis 1b**: Internationalization breadth has a positive association with the innovation of family firms.

#### The moderating role of SEW.

Based on the previous analysis, we propose that the role of SEW in shaping the relationship between internationalization and innovation in family firms may be examined from two distinct aspects: the intention for family control, and transgenerational succession.

***The moderating role of family control***. Firstly, internationalization is usually highly professional and complex, requiring specialized knowledge, advanced skills, and a deep understanding of external markets and institutional environments [40]. Family firms that prioritize preserving family control tend to appoint family members for the top management roles. Consequently, this practice may restrict the recruitment of talented professionals, as it could potentially threaten the family control over the firm [13]. Therefore, family firms with a strong desire to maintain family control usually lack sufficient professional knowledge, advanced skills, and international market knowledge. This deficiency can lead family firms to make incorrect internationalization strategic decisions and/or make it impossible for them to effectively implement these strategies, hence weakening the effect of internationalization on the innovation of family firms. Furthermore, family firms prioritize family control, which limits their use of external funding because external equity could dilute family control [41]. Therefore, family firms that prioritize family control often face severe financial constraints, which in turn restrict the impact of internationalization on innovation of these firms. We hypothesize:

**Hypothesis 2a:** Family control (FC)_weakens the relationship between internationalization and family firm innovation, such that the relationship between internationalization and innovation is stronger at a low level of FC in family firms.

***The moderating role of transgenerational succession intention***. The intention for transgenerational succession leads the family to emphasize the firm’s long-term survival [27], prompting these firms to prioritize long-term strategic investments such as R&D. This may decrease the impact of internationalization on the innovation of family firms. Furthermore, the intention for transgenerational succession signifies the continuity of family culture within the firm, often posing challenges for non-family managers attempting to integrate into family firms due to differing values and a lack of understanding of the firm’s historical dynamics. This barrier to integration might limit the absorption of external knowledge, ultimately hindering innovation during the internationalization process. We hypothesize:

**Hypothesis 2b:** Transgenerational succession intention (TSI) weakens the relationship between internationalization and family firm innovation, such that the relationship between internationalization and innovation is stronger at a low level of TSI in family firms.

#### The moderating role of institutional environment.

The macro-institutional environment significantly influences the internationalization strategies of family firms [16]. During China’s economic transition, significant differences in institutional environment among different regions were observed. We posit that well-developed institutional environment magnifies the impact of internationalization on innovation in family firms. A well-developed institutional environment is typically characterized by little government intervention, robust market mechanisms, and effective legal enforcement, which facilitate efficient resource allocation, smooth information flow, and strong intellectual property protection. In these regions, family firms are likely to obtain scarce resources (e.g., financial capital, human capital, knowledge, and information) more effectively [42] to support internationalization and innovation. Furthermore, the high level of information transparency and market competition in these regions enable family firms to better evaluate and select possible international partner, thereby increasing their chances of acquiring advanced technologies. This environment also promotes knowledge sharing and transformation, allowing family firms to effectively absorb external knowledge during the internationalization process and apply it to their innovation initiatives. Additionally, strong intellectual property protection reduces the risk of technology leakage, enabling family firms to innovate and develop new products in the international market. In contrast, a weak institutional environment is characterized by excessive government intervention, underdeveloped market mechanisms, and inefficient legal enforcement. These deficiencies result in inefficient resource allocation, limited information flow, and weak intellectual property protection, making it more difficult for family firms to get crucial resources through market mechanisms. Furthermore, a low level of information transparency and limited market competition increase uncertainty for family firms in evaluating and selecting international partners, reducing their chances of acquiring advanced technologies. Additionally, weak intellectual property protection raises the risk of technology leakage, making family firms more cautious to innovate in the international market. Based on the above arguments, we hypothesize:

**Hypothesis 3:** Well-developed institutional environment enhances the relationship between internationalization and family firm innovation, such that the relationship between internationalization and innovation is stronger in regions with a well-developed institutional enviornment.

## Methodology

### Sample and data

We conducted an analysis of survey data from Chinese private enterprises obtained between August and October 2020. The survey was performed by Institute of Chengdu-Chongqing Economic Zone Development at Chongqing Technology and Business University. The questionnaire was disseminated in both eastern China (including Shanghai, Zhejiang, Fujian, and Guangdong,) and western China (including Chongqing, Qinghai, Yunnan, and Shaanxi). These regions were chosen to reflect the most developed and least developed sectors of China’s private economy. The survey was designed without limitations on firm size, age, or industry, ensuring that the sample was representative. The initial sample consists of private enterprises engaged in international commerce (e.g., exporting and OFDI activities). The respondents are key decision-makers in their firms. 350 questionnaires were distributed, and 342 were collected, yielding a response rate of 97.71%. These samples included both family and non-family firms. Family firms were identified based on having at least 50% of family ownership and incorporating at least one family member in a key management position [43]. This resulted in a final sample of 253 family firms for our studies, representing an effective rate of 74.0%. The samples can be categorized as follows: Zhejiang (40.3%), Shanghai (5.5%), Fujian (2.4%), Guangdong (1.6%), Chongqing (34.8%), Qinghai (7.5%), Shaanxi (4.7%), Yunnan (3.2%). The sectors of family firms represented include manufacturing (76.7%), services (17.2%), agriculture, forestry, animal husbandry, and fisheries (4.4%), and construction (1.6%). The maximum number of countries in which a family firm operates is 52, the minimum is 1 and average is 2.53. The ratio of family firms involved in exporting compared to outward foreign direct investment (OFDI) was 239–35. Out of the participants engaged in OFDI, 8 family firms built manufacturing facilities, 10 engaged in acquisitions, 21 created sales organizations abroad, and 5 set up research and development (R&D) facilities overseas.

Most family firms in China are classified as small and medium-sized enterprises (SMEs), and there is scarcity of public data regarding these non-listed family firms. We consider the data related to those firms are reliable for two reasons: first, we compared with the data from the questionnaire survey and from database of Chinese listed companies for the listed companies in samples, demonstrating that the sample data is reliable; second, we conducted four questionnaire surveys for the majority of sampled firms from 2008–2020 year, demonstrating that the sample data is reliable by analyzing four survey results. Due to the absence of the internationalization data from sampled firms in four questionnaire surveys, we were unable to perform a panel-data analysis.

### Measures

#### Dependent variables.

The research utilizes R&D intensity as an indicator of a firm’s innovation [27,44], with R&D intensity measured by the proportion of R&D expenditure to total sales revenue in 2019 year. The survey specifies seven categories: less than 0.5%, 0.5% to 1%, 1% to 2%, 3% to 5%, 6% to 10%, 11% to 15%, and over 15%, assigning values from 1 to 7 to those seven categories respectively.

#### Independent variables.

Most family firms in China are classified as SMEs, with few foreign investments. Internationalization depth (Depth) is calculated as the ratio of export sales to total sales in 2019 year. Internationalization breadth (Breadth) is calculated as natural logarithm of the number of foreign countries where a firm operates in 2019 year.

#### Moderating variable.

Distinguishing between restricted and extended SEW priorities [14], and drawing on the works of Berrone et al. [14] and Vandekerkhof et al. [45], this research measures SEW using eight specific questions. The initial four questions evaluate the extent of family influence and control within the firm, known as family control (FC, restricted SEW), while the remaining four questions measure the intention for transgenerational succession (TSI, extended SEW). Respondents are asked to indicate the importance they attach to each item on a 5-point Likert scales (1 = totally unimportant, 5 = extremely important). Reliability test yielded Cronbach’ alpha coefficient of 0.892 for the overall index, 0.854 for FC and 0.854 for TSI, demonstrating acceptable reliability. The average scores for FC and TSI are used in the analysis.

**The measurement items for family control (FC) are as follows**:

(1) The family members own the majority of the shares;
(2) The family members have influence over the company’s strategic decisions;
(3) Most executive positions are held by family members;
(4) The purpose is to preserve family control and the business’s independence.

**The measurement items for transgenerational succession intention (TSI) are as follows**:

(5) The goal is to maintain family traditions and the business’s familial nature;
(6) The aim is to generate and preserve employment opportunities for the family;
(7) It is unlikely that the family members would contemplate selling the family business;
(8) The goal is to successfully transfer the business to the next generation of family members.

The institutional Environment (IE) is measured by the marketization index of China’s provinces or municipalities [46]. Many scholars now utilize this index as a substitute variable for the institutional environment in each province or municipality in China [47].

#### Control variables.

In line with prior research, we controlled for a variety of characteristics that could influence family firm innovation. Firstly, we controlled for firm size and firm age, which are the proxy of firms’ accumulated knowledge and experience [48]. Larger firms have more resources to support new initiatives. Firm size (Size), is measured by the natural logarithm of staff count in 2019 year. Firm age (Age), which reflects the experience and the learning accumulated over time, is measured by the natural logarithm of the number of years since the firm was founded [49]. Innovation levels differ by industry, we also controlled for industry-level influences using a dummy variable (Manufacturing), which was set to 1 for the manufacturing industry and 0 otherwise. Furthermore, we used prior firm performance as an indicator for firm capability of innovation input [27]. Prior firm performance (Performance) is measured by asking respondents to compare their firm’s performance to that of domestic competitors during the past three years, with a focus on sales growth, profit growth rate, market share growth rate, and return on total assets. Responses are recorded on a 5-point Likert scale (1 = very bad, 5 = very good), demonstrating good reliability [50]. Family firms commonly recruit external CEOs with international experience to gain access to technology [50], hence we controlled for the firm leader’s international experience (FIE), with 1 if the firm’s owners or senior managers have engaged in abroad education, life, work, or business trip, and 0 otherwise. Additionally, a firm leader’s political connections might facilitate the acquisition of regulatory resources and policy support [51], thereby influencing its innovation. Thus we also controlled for the firm leader’s political ties (Political), with 1 if the firm’s owners or senior managers are deputy of the People’s Congress or a member of the Chinese People’s Political, and 0 otherwise.

## Results

### Descriptive statistics and correlation analysis

Table 1 reveals a negative and significant correlation between innovation and internationalization depth (p < 0.01). Significant correlations are also observed between innovation and control variables, such as firm size, prior firm performance, and leader’s political ties in family firms, confirming the need to control for these variables.

**Table 1:** Descriptive statistics and correlation coefficient.

Variables	Mean	SD	1	2	3	4	5	6	7	8	9	10	11	12
1. R&D	2.932	1.780	1											
2. Depth	47.254	38.786	−0.183^**^	1										
3. Breadth	1.260	0.968	0.077	0.338^***^	1									
4. FC	3.525	0.862	−0.004	0.123^**+**^	0.122^**+**^	1								
5. TSI	3.540	0.877	0.074	0.217^***^	0.120^**+**^	0.635^***^	1							
6. IE	8.177	2.038	0.053	0.348^***^	0.160^*^	0.150^*^	0.223^***^	1						
7. Size	4.101	1.570	0.143^*^	−0.332^***^	0.056	−0.108^**+**^	0.012	−0.256^***^	1					
8. Age	2.242	0.696	−0.016	−0.037	0.242^***^	−0.058	0.111^**+**^	−0.060	0.383^***^	1				
9. Manufacturing	0.767	0.424	0.043	0.034	0.114^**+**^	−0.049	0.028	0.199^**^	0.228^***^	0.097	1			
10. Performance	3.286	0.733	0.126^*^	0.118^**+**^	0.132^*^	0.038	0.074	0.087	0.081	−0.028	−0.025	1		
11. FIE	0.730	0.445	0.102	0.037	0.145^*^	−0.062	0.043	−0.025	0.058	0.139^*^	−0.014	0.112^**+**^	1	
12. Political	0.198	0.400	0.121^+^	−0.197^**^	0.051	−0.183^**^	−0.085	−0.385^***^	0.383^***^	0.164^**^	−0.054	0.125^*^	0.074	1

**Notes: +**p < 0.10, ^*^p < 0.05, ^**^p < 0.01, ^***^p < 0.001; SD, standard deviation.

### Test of hypotheses

Using the Ordinary Least Squares (OLS) regression model to present regression results, Model 1 in Table 2 serves as the baseline model, comprising only control variables. To examine the hypothesized moderating effects, interaction terms are created between SEW and internationalization (depth and breadth), as well as between the institutional environment and internationalization (depth and breadth), using mean-centered variables to alleviate multicollinearity [52]. The variance inflation factor (VIF) for all variables shows the highest VIF value is less than 2, far below the threshold, indicating multicollinearity is not a significant concern in this study [53].

**Table 2:** Results of internationalization on family firm innovation (R&D intensity).

Variables	Model 1	Model 2	Model 3	Model 4	Model 5
Constant	0.971(0.928)	1.155(0.902)	1.988^*^(0.914)	1.834^*^(0.907)	1.371(0.927)
Size	0.158^+^(0.091)	0.017(0.093)	−0.009(0.091)	0.022(0.091)	−0.016(0.091)
Age	−0.261(0.189)	−0.283(0.186)	−0.305^+^(0.182)	−0.370^*^(0.183)	−0.282(0.182)
Manufacturing	0.069(0.303)	0.095(0.291)	0.122(0.284)	0.132(0.285)	0.008(0.288)
Performance	0.173(0.159)	0.237(0.154)	0.165(0.151)	0.199(0.151)	0.279^+^(0.153)
FIE	0.363(0.264)	0.392(0.254)	0.395(0.248)	0.395(0.249)	0.353(0.249)
Political	0.595^+^(0.349)	0.577^**+**^(0.336)	0.620^+^(0.327)	0.706^*^(0.330)	0.519(0.330)
FC	0.020(0.181)	−0.054(0.175)	−0.201(0.177)	−0.024(0.171)	−0.053(0.171)
TSI	0.071(0.178)	0.238(0.174)	0.258(0.170)	0.086(0.176)	0.281(0.171)
IE	0.073(0.066)	0.111^+^(0.064)	0.113^+^(0.063)	0.117^**+**^(0.063)	0.075(0.066)
Depth		−0.017^***^(0.004)	−0.016^***^(0.003)	−0.016^***^(0.003)	−0.015^***^(0.003)
Breadth		0.275^*^(0.132)	0.296^*^(0.129)	0.280^*^(0.129)	0.313^*^(0.131)
Depth×FC			−0.007^*^(0.003)		
Breadth×FC			−0.375^*^(0.151)		
Depth×TSI				−0.007^*^(0.003)	
Breadth×TSI				−0.348^*^(0.155)	
Depth×IE					−0.006^**^(0.002)
Breadth×IE					0.199^**^(0.064)
*R* ^*2*^ *Adjusted R* ^*2*^ *F* *N*	0.064 0.026 1.685 231	0.151 0.108 3.542 231	0.201 0.153 4.204 231	0.197 0.149 4.092 231	0.198 0.150 4.125 231

**Notes:** +p < 0.10, *p < 0.05, **p < 0.01, ***p < 0.001; Standard errors in parentheses.

### Internationalization and family firm innovation

To test Hypotheses 1a and 1b, we first run a baseline model (Model 1) incorporating only control variables, and then run Model 2 including two independent variables (i.e., internationalization depth and breadth). The results presented in Table 2 show a negative and significant association between internationalization depth and family firm innovation (β = −0.017,p < 0.001), as well as a positive and significant association between internationalization breadth and family firm innovation (β = 0.275, p < 0.05). These results were still robust considering the moderating effects of SEW and the institutional environment in the home country (see Model 3, 4 and 5), Thus, Hypotheses 1a and 1b are supported.

### The moderating role of SEW

We run Model 3 and 4 to test Hypothesis 2 concerning the moderating effects of SEW on the relationship between internationalization and innovation in family firms.

In Model 3 of Table 2, both interactions between family control and internationalization depth (Depth×FC), and interaction between family control and internationalization breadth (Breadth×FC) were found to be negatively and significantly related to the innovation of family firms (β = −0.007, p < 0.05; β = −0.375, p < 0.05). In Model 4 of Table 2, both interactions between transgenerational succession intention and internationalization depth (Depth×TSI), and interaction between transgenerational succession intention and internationalization breadth (Breadth×TSI), were found to be negatively and significantly related to the innovation of family firms (β = −0.007, p < 0.05; β = −0.348, p < 0.05).

**Fig 2:**
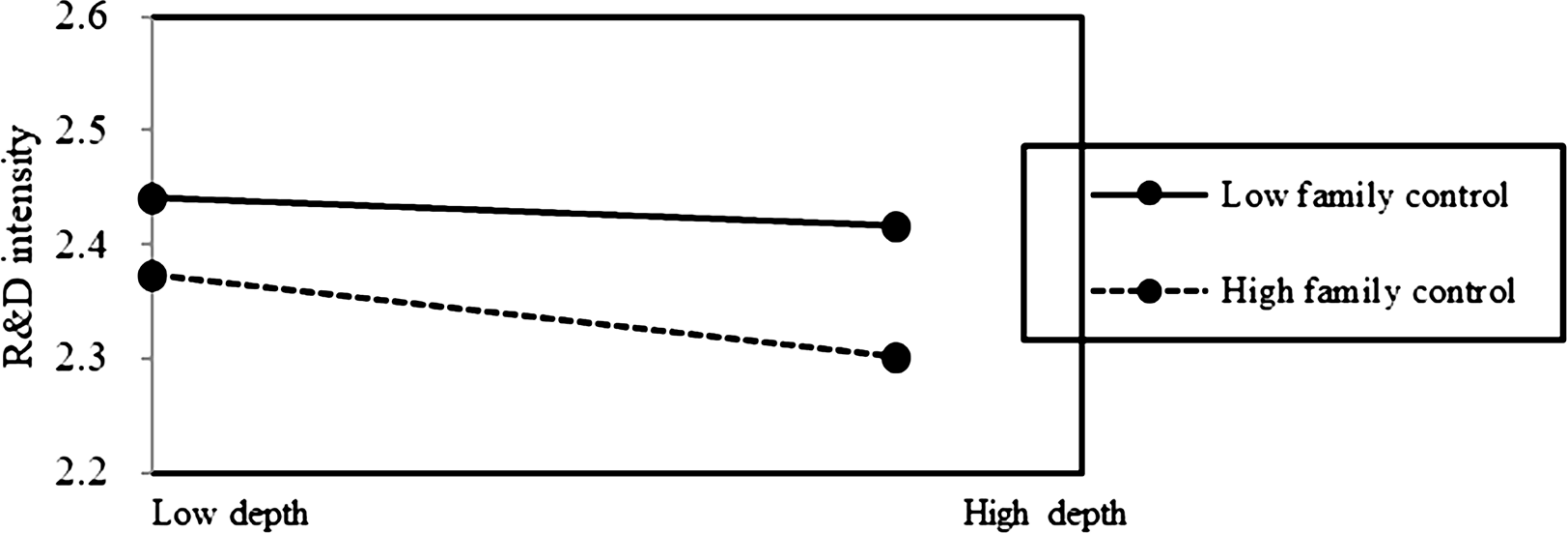
The moderating role of SEW on internationalization depth-innovation relationship.

**Fig 3:**
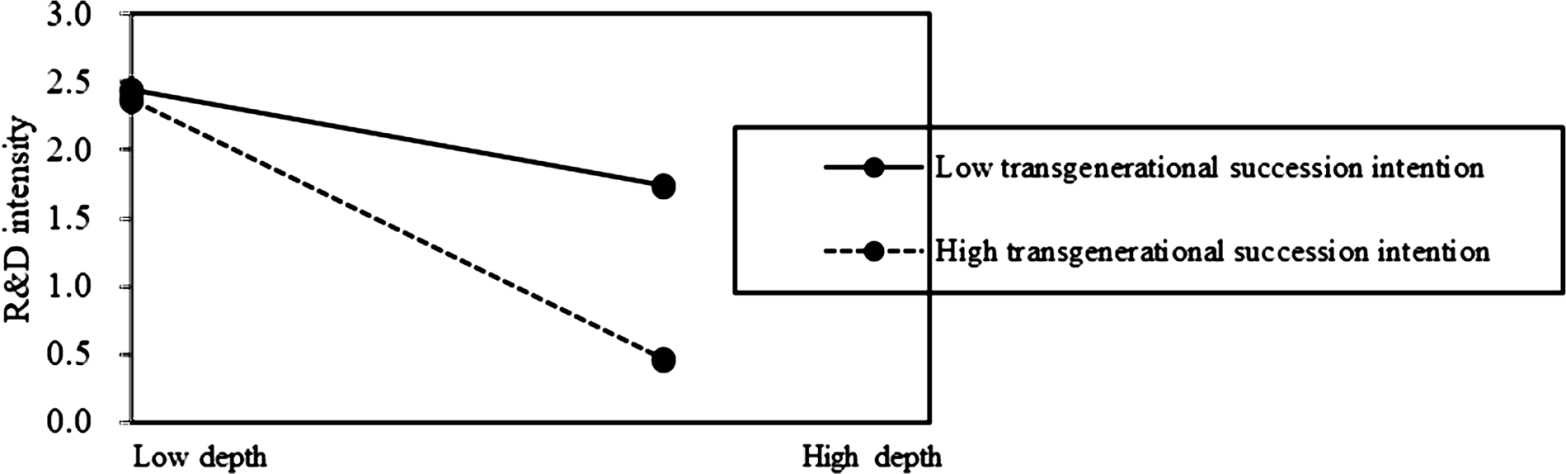
The moderating role of SEW on internationalization depth-innovation relationship.

**Fig 4:**
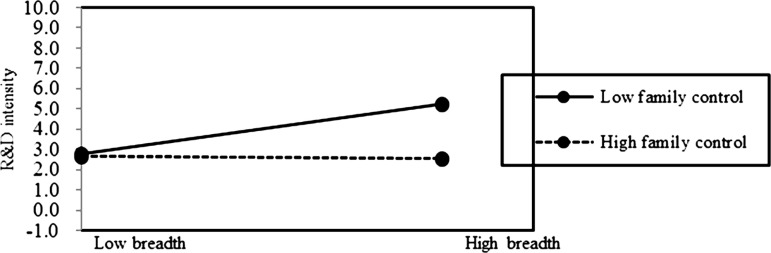
The moderating role of SEW on internationalization breadth-innovation relationship.

**Fig 5:**
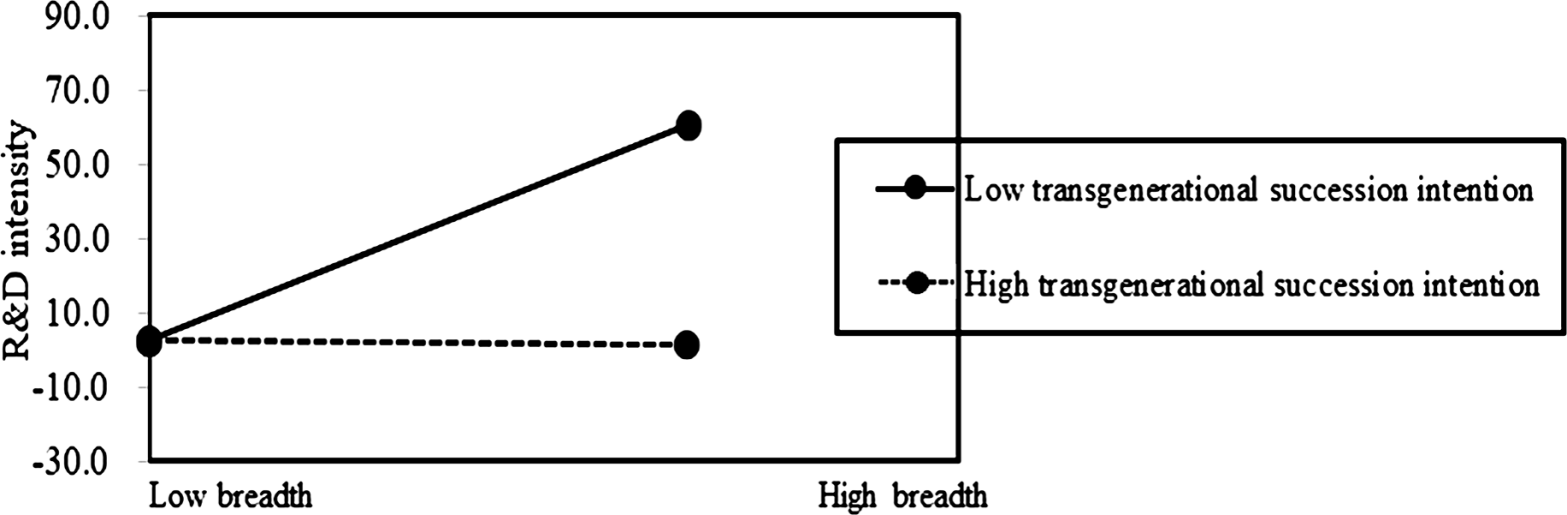
The moderating role of SEW on internationalization breadth-innovation relationship.

Based on the above results, Hypotheses 2b is supported. It indicates that the preserving family control (FC) does not mitigate the negative impact of internationalization depth on family firm innovation. On the contrary, the negative association of internationalization depth on innovation is pronounced at a high level of FC in family firms (Figs 2 and 3). Additionally, it indicates that the positive effect of internationalization breadth on innovation is pronounced at a low level of TSI in family firms (Figs 4 and 5). This suggests that prioritizing SEW can foster risk-averse behavior in family firms, leading them to avoid deep and extensive involvement in international markets, thus enhancing the negative association of internationalization depth on family firm innovation, but weakening the positive association of internationalization breadth on family firm innovation.

### The moderating role of institutional environment

We run Model 5 to test Hypothesis 3 concerning the moderating effects of regional institutional environment in home country on the relationship between internationalization and innovation in family firms.

**Fig 6:**
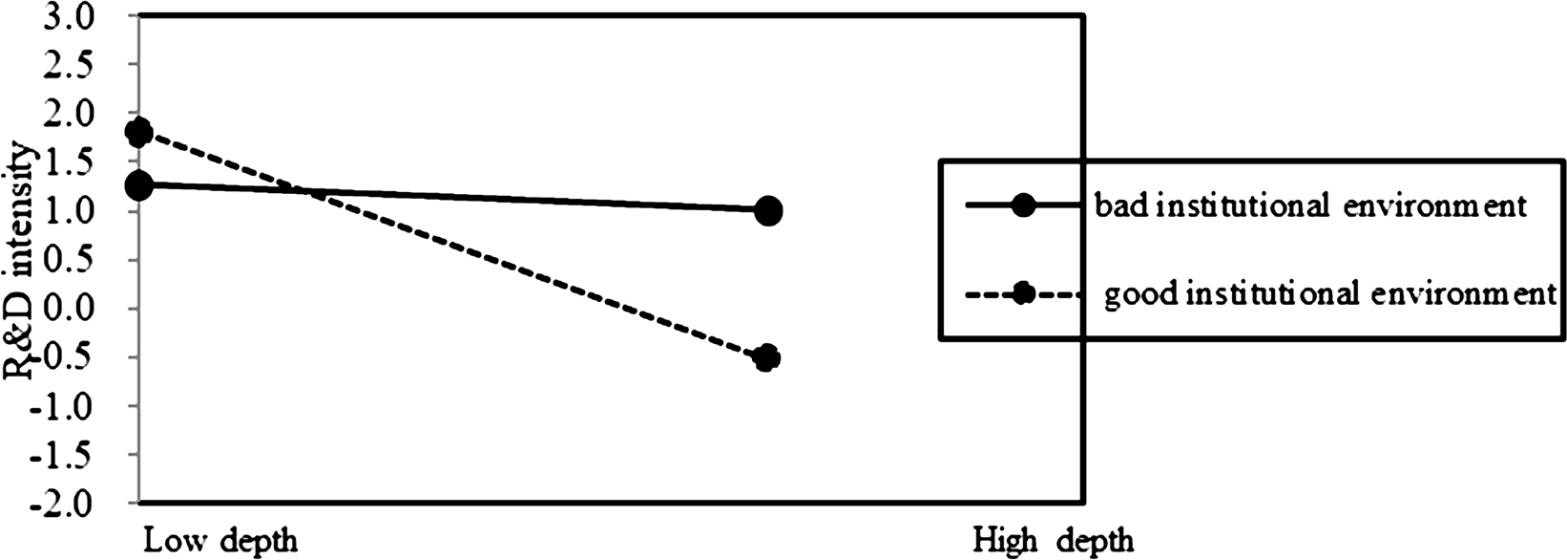
The moderating role of institutional environment on internationalization depth-innovation relationship.

**Fig 7:**
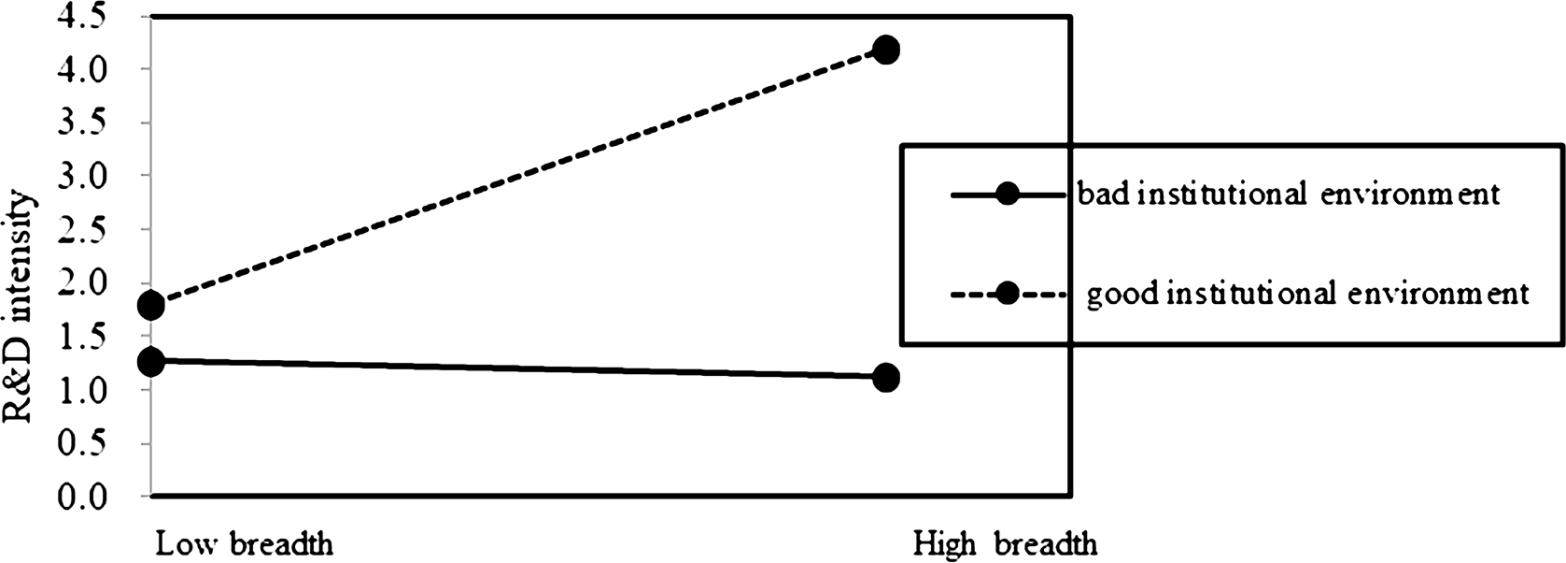
The moderating role of institutional environment on internationalization breadth-innovation relationship.

In Model 5 of Table 2, the interaction of institutional environment with internationalization depth (Depth×IE) had a negative association with innovation (β = −0.006, p < 0.01), whereas the interaction of institutional environment with internationalization breadth (Breadth×IE) had a positive association with innovation (β = 0.199, p < 0.01). Hypothesis 3 is supported. The findings suggest that with improvements in regional institutional environment in home country, the negative impact of internationalization depth on family firm innovation intensifies, whereas the positive impact of internationalization breadth on family firm innovation strengthens (Figs 6 and 7).

### Robustness test

The robustness of our findings has been assessed in two ways. First, the number of patent applications was utilized as an objective indicator of firm innovation, providing a tangible measure of innovative progress [54]. In our sample, most family firms have zero patent applications. To measure the number of patent applications, we used the natural logarithm of the patent counts adding 1 in 2019 year. The results of this analysis are displayed in Table 3.

**Table 3:** Result of internationalization on family firm innovation (Number of patent applications).

Variables	Model 1	Model 2	Model 3	Model 4	Model 5
Constant	−1.004^+^(0.549)	−0.864(0.553)	−0.384(0.560)	−0.308(0.546)	−0.717(0.591)
Size	0.255^***^(0.054)	0.230^***^(0.056)	0.214^***^(0.055)	0.232^***^(0.054)	0.199^***^(0.056)
Age	0.012(0.112)	−0.027(0.114)	−0.048(0.111)	−0.092(0.111)	−0.006(0.112)
Manufacturing	0.303^+^(0.179)	0.299^+^(0.179)	0.314^+^(0.174)	0.332^+^(0.172)	0.244(0.178)
Performance	0.055(0.094)	0.048(0.094)	0.005(0.092)	0.019(0.091)	0.099(0.095)
FIE	0.190(0.156)	0.180(0.156)	0.188(0.152)	0.185(0.150)	0.167(0.154)
Political	0.310(0.206)	0.274(0.206)	0.302(0.201)	0.376^+^(0.199)	0.264(0.204)
FC	0.115(0.107)	0.083(0.107)	0.001(0.109)	0.105(0.103)	0.087(0.106)
TSI	−0.009(0.106)	0.033(0.107)	0.048(0.104)	−0.090(0.106)	0.055(0.106)
IE	−0.047(0.039)	−0.039(0.040)	−0.037(0.039)	−0.036(0.038)	−0.073(0.046)
Depth		−0.004^+^(0.002)	−0.004^+^(0.002)	−0.003(0.002)	−0.004^+^(0.002)
Breadth		0.137^+^(0.079)	0.151^+^(0.077)	0.143^+^(0.076)	0.183^*^(0.080)
Depth×FC			−0.006^**^(0.002)		
Breadth×FC			−0.191^*^(0.093)		
Depth×TSI				−0.005^*^(0.002)	
Breadth×TSI				−0.302^**^(0.094)	
Depth×IE					−0.002^*^(0.001)
Breadth×IE					0.079^*^(0.035)
*R* ^*2*^ *Adjusted R* ^*2*^ *F* *N*	0.227 0.195 7.208^***^ 231	0.244 0.206 6.418^***^ 231	0.2922 0.250 6.896^***^ 231	0.311 0.270 7.540^***^ 231	0.275 0.232 6.330^***^ 231

**Notes:** +p < 0.10, ^*^p < 0.05, ^**^p < 0.01, ^***^p < 0.001; Standard errors in parentheses.

Internationalization depth was negatively related to family firm innovation, whereas internationalization breadth was positively related to family firm innovation. Furthermore, the interactions between family control with internationalization depth and breadth were negatively related to family firm innovation. Similarly, the interactions between transgenerational succession intention with depth and breadth were negatively related to family firm innovation. Additionally, the interaction institutional environment with internationalization depth was negatively related to family firm innovation, whereas the interaction with institutional environment and internationalization breadth was positively related to family firm innovation. These findings confirm the consistency and robustness of our initial results.

Second, we use an alternative definition for family businesses to robust our results reported in Table 4. The definition used for family business is as follows: family businesses are those with at least 50% family ownership. The results suggest that internationalization depth had a negative and significant impact on innovation, whereas internationalization breadth had a positive and significant impact on innovation. Family control, and transgenerational succession intention negatively moderated the relationship between internationalization and innovation. Institutional environment negatively moderated the relationship internationalization depth and innovation, whereas they positively moderated the relationship internationalization breadth and innovation. The robustness test results indicate consistency in our primary findings.

**Table 4:** Results of internationalization on family firm innovation (R&D intensity).

Variables	Model 1	Model 2	Model 3	Model 4	Model 5
Constant	0.391(0.868)	0.698(0.840)	1.407^+^(0.848)	1.216(0.840)	0.924(0.863)
Size	0.187^*^(0.084)	0.069(0.084)	0.044(0.083)	0.067(0.083)	0.059(0.083)
Age	−0.234(0.178)	−0.279(0.174)	−0.315^+^(0.171)	−0.367^*^(0.173)	−0.273(0.170)
Manufacturing	−0.017(0.282)	−0.081(0.270)	−0.056(0.264)	−0.057(0.265)	−0.163(0.267)
Performance	0.241(0.148)	0.266^+^(0.144)	0.188(0.142)	0.215(0.142)	0.293^*^(0.142)
FIE	0.307(0.247)	0.306(0.237)	0.312(0.232)	0.290(0.233)	0.276(0.232)
Political	0.607^+^(0.317)	0.539^+^(0.303)	0.593^*^(0.296)	0.656^*^(0.300)	0.447(0.299)
FC	−0.024(0.168)	−0.101(0.161)	−0.227(0.162)	−0.081(0.158)	−0.093(0.157)
TSI	0.059(0.164)	0.205(0.159)	0.236(0.155)	0.131(0.158)	0.214(0.156)
IE	0.129^*^(0.061)	0.173^**^(0.059)	0.179^**^(0.058)	0.179^**^(0.058)	0.143^*^(0.061)
Depth		−0.016^***^(0.003)	−0.015^***^(0.003)	−0.015^***^(0.003)	−0.013^***^(0.003)
Breadth		0.330^**^(0.113)	0.337^**^(0.111)	0.330^**^(0.112)	0.303^**^(0.112)
Depth×FC			−0.007^*^(0.003)		
Breadth×FC			−0.333^*^(0.132)		
Depth×TSI				−0.008^**^ (0.003)	
Breadth×TSI				−0.220^+^ (0.119)	
Depth×IE					−0.005^**^(0.002)
Breadth×IE					0.177^**^(0.058)
*R* ^*2*^ *Adjusted R* ^*2*^ *F* *N*	0.0886 0.052 2.535^**^ 253	0.177 0.140 4.724^***^ 253	0.222 0.179 5.235^***^ 253	0.216 0.174 5.070^**^ 253	0.219 0.177 5.163^***^ 253

**Notes:** +p < 0.10, ^*^p < 0.05, ^**^p < 0.01, ^***^p < 0.001; Standard errors in parentheses.

## Discussion and conclusions

Although internationalization is regarded as an important opportunity for emerging market firms to gain knowledge and update inventive capacities, the influence of internationalization on innovation in a family firm remains poorly understood. In this research, we investigate the effects of internationalization depth and breadth on family firm innovation, as well as the moderating influences of SEW and the home country’s regional institutional environment. Drawing on the data of 253 family firms in China, this study produces three significant conclusions, as well as noteworthy contributions to the field in numerous areas.

Firstly, our empirical findings reveal that there are considerable disparities in the depth and breadth of internationalization on innovation for family firms. Internationalization depth is negatively and significantly related to the innovation of family firms, whereas internationalization breadth is positively and significantly related to the innovation of family firms. To guarantee robustness, we validate our findings by substituting the assessment of R&D intensity with the number of patent applications and using other family business definitions. The primary reasons are as follows. High depth implies that family firms are exposed to more concentrated countries with fewer challenges, which may limit their opportunities to gain new knowledge in other market. However, such knowledge may be highly valuable, scarce, inimitable, and irreplaceable, helping family firms maintain a competitive advantage. Nevertheless, this lack of exposure to diverse markets may result in a shortage of new perspectives and ideas for innovation in family firms. High breadth suggests that family firms possess broad relational networks, which allow them to exploit knowledge spillovers from numerous markets [11], so increasing their innovative capability. In contrast to previous research that has shown a positive and significant relationship between internationalization depth and family firm innovation [7–9], our study reveals both negative and positive effects of internationalization depth and breadth on the innovation of family firms, Therefore, our findings also make a valuable contribution to the existing literature on the correlation between internationalization and innovation with a family firm context.

Second, our empirical study indicates that the impact of internationalization on family firm innovation is contingent upon the specific SEW goal that the firm aim to accomplish, which has not been explored in the previous literature [7–9]. In particular, the negative influence of internationalization depth on innovation is stronger with higher restricted SEW (FC), whereas the positive influence of internationalization breadth on innovation is stronger with lower extended SEW (TSI) in family firms. According to Chrisman and Patel [27], family firms tend to avoid making decisions that prioritize economic rewards over SEW. The empirical evidence we present partially support for this argument. Family firms, for example, are inclined to refrain from seeking extensive access to international markets in order to protect their SEW, which hinders the enhancement of their innovative capabilities. Our study also reveals the presence of the ‘dark side’ of certain SEW dimensions [18]. We discovered that TSI has a negative moderating impact on the positive association between internationalization breadth and innovation in family firms, that is, TSI considerably diminishes the positive impact of internationalization breadth on the innovation of family firms. The inclusion of the level of SEW preservation in this paper is as a response to the request made by multiple researches to consider emotions when examining the organizational processes of family firms [13,45].

Third, our empirical study indicates that the impact of internationalization on innovation in family firms is contingent on the regional institutional environment in the home country. Specifically, when the institutional environment improves, the negative impact of internationalization depth on family firm innovation becomes more pronounced, whereas the positive impact of internationalization breadth on family firm innovation becomes more pronounced. Therefore, our study contributes to the existing empirical evidence that highlights the important influence of institutional contexts on the internationalization of family firms [16,39].

Our findings provide interesting practical implications for family firms. First, our study shows internationalization breadth promotes innovation, whereas internationalization depth hinders innovation. As a result, family firms should actively expand their international market scope, but should be cautious when evaluating investments in existing international markets. Family firms, particularly those in low-technology industries, should avoid substantial integration into foreign markets. Second, our findings show that the preservation of family SEW has an impact on the connection between internationalization and innovation in Chinese family firms.

As a result, family firms must strike a delicate balance between meeting both business and family demand to assure that internationalization breadth has a positive impact on family firm innovation. Finally, government agencies should enhance the institutional framework to facilitate the international expansion of family firms.

This study has several limitations. First, we utilize one metric to judge the level of internationalization. Future study could look into internationalization speed and entry mode. Second, we just utilize two dimensions to define SEW. Future research should look at the various components of SEW to give a consistent measure of affective endowments in family firms. Finally, future research can employ panel data to better understand the link between internationalization and innovation in family firms.
